# Retraction: STEAP1 facilitates metastasis and epithelial–mesenchymal transition of lung adenocarcinoma via the JAK2/STAT3 signaling pathway

**DOI:** 10.1042/BSR20193169_RET

**Published:** 2025-11-27

**Authors:** Shu-fen Huo, Wen-li Sheng, Min Yu, Xiao-ping Ren, Hong-xia Wen, Chun-yan Chai, Li Sun, Ke Hui, Ling-hua Liu, Sheng-hong Wei, Xiao-xiao Wang, Yi Wang, Ying-xuan Tian

**Affiliations:** 1 Second Department of Respiratory Medicine, Shannxi Provincial People’s Hospital, Xi’an, 710068, China; 2 Department of Medical Oncology, Shannxi Provincial People’s Hospital, Xi’an, 710068, China; 3 The First Ward, Shannxi Provincial People’s Hospital, Xi’an, 710068, China

This article is being retracted from *Bioscience Reports* at the request of the authors. After receiving notice from the authors and from a reader, the Editorial Board has been made aware of errors in the pictures of this article and multiple similarities between articles published in other journals. Specifically, these include the following:

– In Figure 3C, similarities are apparent between the migration vector and invasion vector panels.
– Similarities between this article and PMC5354784 (10.18632/oncotarget.14283) by Chen et al. 2016
– Figure 3B H1299/NC panel with PMC5354784 (10.18632/oncotarget.14283) Figure 6F Invasion/si-Control panel
– Figure 3B H1299/shRNA panel with PMC5354784 (10.18632/oncotarget.14283) Figure 6F Migration/si-Control panel
– Figure 5C NC+ADZ1480/invasion panel with PMC5354784 (10.18632/oncotarget.14283) Figure 6F Invasion/si-Twiat1 panel
– Similarities between this article and PMC8314571 (10.1186/s12935-021-02098-1) by Li et al. 2021
– Figure 2E cyclinD1 bands with PMC814571 (10.1186/s12935-021-02098-1) Figure 6C IGHG1 bands
– Figure 3D MMP9 middle two bands with PMC814571 (10.1186/s12935-021-02098-1) Figure 6C p-GSJ-3B middle two bands
– Similarities between this article and PMC7885357 (10.1186/s12935-021-01811-4) by Chen et al. 2021
– Figure 3D snail1 bands with PMC7885357 (10.1186/s12935-021-01811-4) Figure 3E Ncadherin bands
– Figure 4A N-cadherin bands with PMC7885357 (10.1186/s12935-021-01811-4) Figure 3E Ki67 bands
– Figure 4A vimentin bands with PMC7885357 (10.1186/s12935-021-01811-4) Figure 3E Vimentin bands
– Figure 4A E-cadherin bands with PMC7885357 (10.1186/s12935-021-01811-4) Figure 3E Ecadherin bands
– Similarities between this article and PMC5029729 (10.18632/oncotarget.8260) by Fan et al. 2016
– Figure 4F GADPH bands with PMC5029729 (10.18632/oncotarget.8260) Figure 6A H2AX bands

The authors maintain different views on the questions raised by the readers. The authors point out that their article was published before Chen et al. 2021 and Li et al. 2021, and they disagree with the above similarities pointed out by readers.

Given that this study was conducted over a decade ago, they state that there may be some imperfections in the process. The authors also frankly admit that there are concerns over the rigor of the paper and apologise for the lack of rigor in some aspects of the paper. The Editorial Board has lost confidence in the article's rigor and agree with the decision of the authors to retract the article.

